# Trait-Based Climate Change Predictions of Vegetation Sensitivity and Distribution in China

**DOI:** 10.3389/fpls.2019.00908

**Published:** 2019-07-12

**Authors:** Yanzheng Yang, Jun Zhao, Pengxiang Zhao, Hui Wang, Boheng Wang, Shaofeng Su, Mingxu Li, Liming Wang, Qiuan Zhu, Zhiyong Pang, Changhui Peng

**Affiliations:** ^1^College of Forestry, Northwest A&F University, Yangling, China; ^2^State Key Laboratory of Urban and Regional Ecology, Research Center for Eco-Environmental Sciences, Chinese Academy of Sciences, Beijing, China; ^3^State Key Laboratory of Hydroscience and Engineering, Department of Hydraulic Engineering, Tsinghua University, Beijing, China; ^4^Institute of Surface-Earth System Science, Tianjin University, Tianjin, China; ^5^Department of Biology Sciences, Institute of Environment Sciences, University of Québec at Montreal, Montreal, QC, Canada

**Keywords:** trait covariations, trait–climate relationships, Gaussian mixture model, vegetation modeling, vegetation sensitivity

## Abstract

Dynamic global vegetation models (DGVMs) suffer insufficiencies in tracking biochemical cycles and ecosystem fluxes. One important reason for these insufficiencies is that DGVMs use fixed parameters (mostly traits) to distinguish attributes and functions of plant functional types (PFTs); however, these traits vary under different climatic conditions. Therefore, it is urgent to quantify trait covariations, including those among specific leaf area (SLA), area-based leaf nitrogen (*N*_area_), and leaf area index (LAI) (in 580 species across 218 sites in this study), and explore new classification methods that can be applied to model vegetation dynamics under future climate change scenarios. We use a redundancy analysis (RDA) to derive trait–climate relationships and employ a Gaussian mixture model (GMM) to project vegetation distributions under different climate scenarios. The results show that (1) the three climatic variables, mean annual temperature (MAT), mean annual precipitation (MAP), and monthly photosynthetically active radiation (mPAR) could capture 65% of the covariations of three functional traits; (2) tropical, subtropical and temperate forest complexes expand while boreal forest, temperate steppe, temperate scrub and tundra shrink under future climate change scenarios; and (3) the GMM classification based on trait covariations should be a powerful candidate for building new generation of DGVM, especially predicting the response of vegetation to future climate changes. This study provides a promising route toward developing reliable, robust and realistic vegetation models and can address a series of limitations in current models.

## Introduction

Vegetation determines the fluxes of energy and water and the variation in CO_2_ to and from terrestrial ecosystems (van Bodegom et al., 2014). As an essential tool in vegetation modeling, dynamic global vegetation models (DGVMs, see Prentice and Cowling, 2013) typically use fixed values to distinguish differences in the structure and function of plant functional types (PFTs) and do not permit these traits to vary or adapt in time and space. However, these traits not only vary but also generally show more variation within PFTs than between PFTs (Cunningham and Read, 2002; van Bodegom et al., 2012). A growing number of studies have rejected PFT classifications because of their insufficiency in describing continuous variation in traits in nature and its low accuracy in modeling ecological processes (van Bodegom et al., 2012; Pavlick et al., 2013; Yang et al., 2015). Ecologists and modelers have attempted to find new methods to improve or replace the PFT framework in DGVMs. One of the most feasible methods is using the relationships between plant functional traits and climate to replace the fixed values of PFTs. Therefore, it is urgent to discuss trait covariation (providing continuous trait variation for ecological processes in the next generation of trait-based DGVM) and the relationships between traits and vegetation types (an important output of trait-based DGVM), especially under future climate scenarios.

Considerable progress has been achieved toward improving the framework of current DGVMs or modeling vegetation distributions based on trait–climate relationships. To overcome the limitations that arise due to fixed or constant traits in PFTs, Verheijen et al. (2013) allowed three key functional traits to vary within PFTs via trait–climate relationships, which enabled more variation in vegetation responses to be included in DGVMs. Pavlick et al. (2013) simulated the performances of a large number of random plant growth strategies, each depicted by a set of 15 traits that represent various ecosystem functions including carbon allocation, ecophysiology, phenology and vegetation dynamics. Similarly, Scheiter et al. (2013) presented a trait- and individual-based vegetation model that permitted individual plants to adopt a combination of trait values. Another individual- and trait- based DGVM, the Lund-Potsdam-Jena managed model with flexible individual traits (LPJmL-FIT), incorporates five traits to describe the performance of trees and achieves a more realistic representation of functional diversity at a regional scale (Sakschewski et al., 2015). Although these approaches have been criticized because some traits are not measurable or because of weak trait–environment relationships, they have provided different perspectives on the construction of new trait-based DGVMs.

It is widely recognized that modeling the vegetation distributions with Gaussian Mixture Models (GMMs) based on plant trait covariations is indispensable for our understanding of climate change impacts on ecosystems and it is a key output of the new generation of DGVMs (Sitch et al., 2008; van Bodegom et al., 2014). This method has been successfully applied in modeling vegetation distributions under historical climate conditions (Yang et al., 2016), although the response of vegetation to future climate scenarios for China based on the method of co-located trait–climate relationships remains unclear.

In this paper, we examined a suite of leaf traits using co-located measurements and quantified the contributions of climate to predict the vegetation distribution in China. Our analysis was based on an extensive data set (Geng et al., 2017; Wang et al., 2018). We focused on three leaf traits, i.e., leaf area index (LAI), specific leaf area (SLA), and leaf nitrogen per unit area (*N*_area_), which together capture many functions of plants, such as carbon investment, photosynthetic ability, and sustaining the leaf temperature (Wright et al., 2004, 2017). We performed multivariate analysis to quantify the co-located trait–climate relationships; then, we trained a GMM with corresponding trait–vegetation relationships; and finally, we examined the response of vegetation to a changing climate. The objectives of this study were to (1) quantify the trait covariations resulting from climate, (2) investigate the relationships between vegetation types and trait covariations, and (3) predict the distribution and response of vegetation to changing climatic conditions.

## Materials and Methods

### Study Area

**FIGURE 1:**
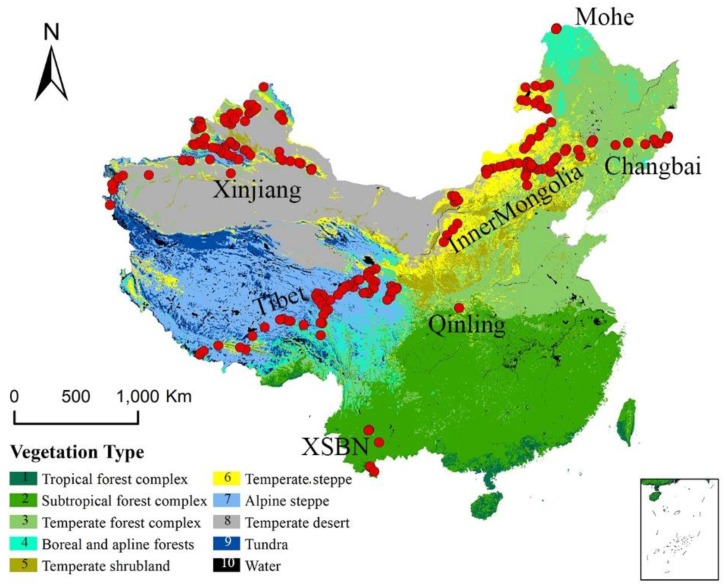
Geographical and climatic coverage of the trait dataset. The individual sites are shown as red dots superimposed on a simplified vegetation map of China; these sites have been grouped into seven named regions (Hou, 2001).

China contains a wide range of vegetation types, from tropical rainforest to boreal coniferous forests and alpine vegetation (Figure 1; Hou, 2001). China is home to more than 33,000 vascular plant species and among the world’s richest countries in terms of plant biodiversity (López-Pujol et al., 2006). Annual average temperatures range from -21.0°C to 26.0°C and increase from north to south. Annual precipitation ranges from 0 to 2250 mm and decreases from southeast to northwest. A steep climate gradient and abundant plant species make China an ideal region to analyze the response of vegetation to changing climates.

### Leaf Traits and Site Distributions

We selected two plant functional traits [SLA (m^2^/kg) and *N*_area_ (g/m^2^)] and one structural trait of plant communities (LAI) in this study. From 2003 to 2013, we collected 1,192 functional trait observations from 580 species across 218 sites (Geng et al., 2017; Wang et al., 2018), which occupied the main climate space in China (Figure 1 and Supplementary Table S2) and the dominate species in the sampling sites were selected. The sampling sites could be classified into seven main regions. The northernmost region was Mohe and the main vegetation types was boreal forest. Sites from Changbai to Inner Mongolia were distributed across an aridity gradient (Prentice et al., 2011). Sites from Inner Mongolia were distributed along the 400 mm equivalent precipitation line (Geng et al., 2017). The northwestern sites were distributed in Mount Altai and Mount Tian in the Xinjiang Autonomous Region. The southwestern sites were located in Xishuangbanna (XSBN) and the main vegetation types are subtropical and tropical forests.

Leaf area (LA) is a key trait that links plant form, function, and environment (Blonder et al., 2012). LA determines light interception and LA differences are associated with variations in within-leaf support investment and determine leaf chemical and structural characteristics (Niinemets et al., 2007). We used continuous records for LAI from 8-day Moderate Resolution Imaging Spectroradiometer (MODIS) data (MOD15A2, 549 periods) to replace the LA; these data were averaged, and the effect of cloud-contaminated or aerosol-contaminated reflectance was removed using the methods proposed by Myneni et al. (2007).

Specific leaf area is the ratio of LA to dry mass and represent a key index variable of the leaf economic spectrum (LES) through its inverse relationship with leaf longevity (Wright et al., 2004). High-SLA leaves have a low investment cost but are also short lived and susceptible to herbivory, while low-SLA leaves are robust but expensive to construct (Coley et al., 1985). *N*_area_ has often been considered an index of photosynthetic capacity, has been shown to vary with climate and is generally high in more arid environments (Wright et al., 2002, 2004, 2005; Prentice et al., 2011; Dong et al., 2017). The average values of SLA and *N*_area_ were calculated for all species in each site without consideration of species abundance.

### Climate Data

For future climates, a set of scenarios known as Representative Concentration Pathways (RCPs) have been widely adopted by climate and ecology researchers to provide a range of possible futures for analyzing vegetation dynamics (Moss et al., 2010; Gao et al., 2016). RCPs were used for new climate model simulations under the framework of the Coupled Model Intercomparison Project Phase 5 (CMIP5) of the World Climate Research Programme (Stocker et al., 2013). Compared with previous scenarios, RCPs consider more changing information needed by policy makers (Moss et al., 2010), and the projected global mean surface temperature increases range from 1.5°C by 2100 for the lowest of the four RCPs (RCP3-PD and RCP2.6) to 4.5°C for the highest one (RCP8.5) (Meinshausen et al., 2011). The lowest, medium and highest RCPs, namely RCP2.6, RCP4.5, and RCP8.5 respectively, were selected in this study (Table 1).

**Table 1:** Information on the RCPs used in this study.

Type	Radiative forcing	Concentration (ppm)	Trends	Model and providing institute	Increase in global mean temperature change for 2081–2100 relative to 1986–2005
RCP2.6	Peak at ∼3 W m^-2^ before 2100 and then decline	Peak at ∼490 CO_2_ equivalents before 2100 and then decline	Peak and then decline	IMAGE, NMP^1^	0.3∼1.7°C
RCP4.5	∼4.5W m^-2^ at stabilization after 2100	∼650 CO_2_ equivalents and then stabilization after 2100	Stabilization without overshoot	GCAM, PNNL^2^	1.1∼2.6°C
RCP8.5	>8.5 W m^-2^ in 2100	>1370 × 10^-6^ CO_2_ equivalents in 2100	Rising	MESSAGE, IIASA^3^	2.6∼4.8°C


*^1^IMAGE, Integrated Model to Assess the Global Environment, Netherlands Environmental Assessment Agency, Netherlands. ^*2*^GCAM, Global Change Assessment Model, Pacific Northwest National Laboratory, United States. ^*3*^MESSAGE, Model for Energy Supply Strategy Alternatives and their General Environmental Impact, International Institute for Applied Systems Analysis, Austria.*

**FIGURE 2:**
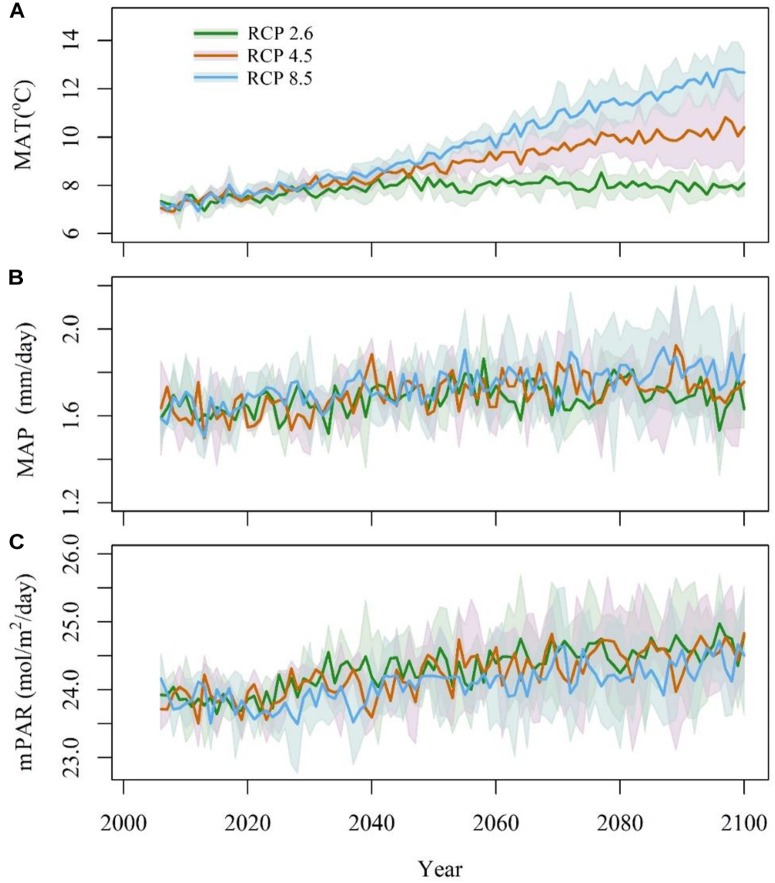
Anomaly of mean annual temperature (**A**, MAT), mean annual precipitation (**B**, MAP), and monthly photosynthetically active radiation (**C**, mPAR) from 2006 to 2100. The blue line stands for RCP8.5, the red line stands for RCP4.5, and the green line stands for RCP2.6. The shadows stand for the 95% confidence intervals.

Three earth system models, IPSL-CM5A-MR, MPI-ESM-MR, and NorESM1-M (Supplementary Table S1) were selected, and the climatic data were downloaded from the CMIP5 website^1^. These data included the mean annual temperature (MAT, °C), mean annual precipitation (MAP, mm/day) and monthly photosynthetically active radiation (mPAR, W ⋅ m^-2^ ⋅ day^-1^). mPAR was calculated by SPLASH v1.0 (Davis et al., 2017) with surface down-welling shortwave radiation (rsds) data. All the climatic data were interpolated into 0.085° × 0.085° with the tools in ANUSPLIN4.4 (Hutchinson and Xu, 2013). To adjust the bias of future climate models, anomalies were calculated as the differences between the model’s data from 2006 to 2014 and historical data from 2006 to 2014. A time series of future climate was produced by subtracting the anomalies (Figure 2). This strategy preserved the continuity and stability between the future model’s data and the historical data (Cramer et al., 2001).

For historical climatic data between 1987 and 2014, the MAT and MAP were derived from 756 meteorological stations and interpolated toa 0.085° × 0.085° resolution (approximately 10 km) using ANUSPLIN4.4. mPAR was extracted from a dataset of reconstructed PAR in China (Tang et al., 2017), which has a high accuracy because it is derived from observed meteorological data and MODIS aerosol optical depth (AOD) data and calibrated by data from 39 ChinaFLUX sites.

### Redundancy Analysis and Trait Prediction

Redundancy analysis (RDA) is a method of extracting the variation in a set of response variables that can be explained by a set of explanatory variables (Borcard et al., 1992). Through calculating eigenvectors and eigenvalues of the covariance matrix of the combination of response variables and explanatory variables, information concerning a number of constrained axes (RDA axes) and unconstrained axes (principal component axes) can be obtained. The constrained axes represent the part of the response variables (trait here) explained by the explanatory variables (climate here), which can be used to predict trait variations under future climate scenarios. Traits were only measured in the dominant species. The SLA and *N*_area_ values were the averaged values for a species, and the LAI value is the average value at the community level. The three climatic variables were represented by the average values at the sampling sites.

### GMM Classification and Simulation Steps

Gaussian functions and their combinations are widely applied in bio-statistics to describe complex distributions and classifications (for algorithm details, see Witte et al., 2007 and Yang et al., 2016). Once Gaussian density distributions are ascertained in the discriminant classification, we can easily obtain the classification probability associated with each class. A GMM is a combination of several Gaussian components that do not require any arbitrary and potentially restrictive assumptions in the form of probability density functions, and it is an effective vegetation classifier in trait-based modeling (Laughlin et al., 2015; Yang et al., 2016).

A GMM was employed and revised in this study (Witte et al., 2007; van Bodegom et al., 2014; Yang et al., 2016). Several steps were taken (Supplementary Figure S1): (a) a RDA was conducted, and trait–climate relationships were built (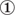); (b) a GMM was trained by the relationships between traits and vegetation types and then validated by natural vegetation maps (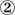
∼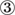);(c) future trait patterns were calculated based on the trait-climate relationships from the RDA (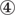); and (d) taking the predicted future traits as inputs, the future vegetation distributions were obtained by the GMM classifier (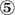
∼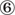).

Climate was one of most important drivers of trait variations (Yang et al., 2019), and trait values were always considered to filter the results of climate changes (Webb et al., 2010). Compared with single trait variations, trait covariation showed a trade-off among traits under changing climatic conditions. On the one hand, trait covariations determined the ecosystem structure and function of ecosystems, which would provide more credible parameters for the ecosystem processes than the fixed ones, on the other hand, trait covariation can simultaneously provide reasonable ranges for each vegetation type, which would help us to classify the trait combinations into different vegetation types using trained GMM.

## Results

### Comparisons of Three Climate Scenarios

The three RCPs differed greatly in temperature, which was highest in RCP8.5, especially after 2050, followed by that in RCP4.5 and RCP2.6 (Figure 2A). The variations in temperature increased considerably before 2050. After 2050, the temperature in RCP8.5 showed a continuous increasing trend. The temperature in RCP2.6 was relatively stable after 2050, and the temperatures in RCP4.5 remain intermediate between those in RCP2.6 and RCP 8.5. Among the three climate models, NorESM1-M had the highest temperature values under RCP2.6 and RCP4.5, followed by IPSL-CM5A-MR and MPI-ESM-MR; however, the temperature in IPSL-CM5A-MR was higher than that in the two other models under RCP8.5.

The annual mean precipitation did not differ greatly among the four climate models (Figure 2B). The annual mean precipitation was 611 mm under RCP2.6, 626 mm under RCP4.5, and 648 mm under RCP8.5. Under the three climatic scenarios, NorESM1-M had the highest annual mean precipitation, followed by MPI-ESM-MR and IPSL-CM5A-MR. The annual mean precipitation increased from 2006 to 2040 and remained relatively stable during 2041–2100. For precipitation, mPAR was relatively stable from 2006 to 2020 and then quickly increased from 2020 to 2030 before finally stabilizing at a relatively high level (Figure 2C). Under RCP2.6 and RCP4.5, NorESM1-M presented the highest mPAR, followed by MPI-ESM-MR and CanESM2. Under RCP8.5, IPSL-CM5A-MR had the highest mPAR, followed by MPI-ESM-MR and NorESM1-M.

### Linking Trait Covariations Into the Prediction of Vegetation Distributions

**FIGURE 3:**
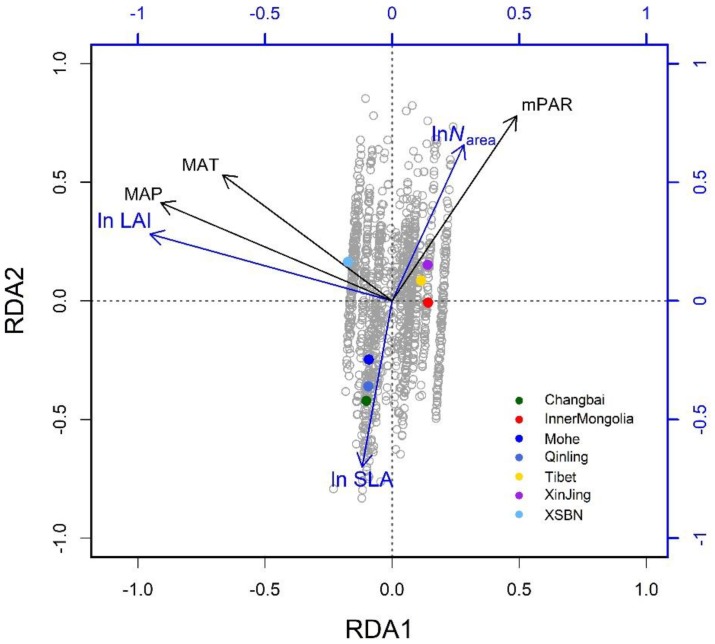
Climate-related trait dimensions from the redundancy analysis: gray circles are species-site combinations and colored dots signify named regions as defined in Figure 1. The traits are SLA, specific leaf area; *N*_area_, leaf nitrogen per unit area; and LAI: leaf area index. The climatic variables are mean annual temperature (MAT), daily precipitation (MAP), and monthly photosynthetically active radiation (mPAR) (in color).

The three climatic variables explained 65% of the trait variation (Table 2). The first two successive RDA axes (Figure 3) described the patterns of trait variation with climate, and showed the between-site patterns of trait covariation imposed by climatic gradients. The first RDA axis was overwhelmingly dominant and related to the gradient of PR from steppe to moist forests. The LAI varied along this gradient, with large leaves characteristic of wetter environments. The second RDA axis accounted for 2% of trait variation and was related to the covariation of the mean growing-season temperature and daily temperature along the latitudinal gradient from the boreal zone to the tropics. Trait variation on this axis resembled that of the LES: warmer, high-irradiance climates were characterized by plants with lower SLA and higher *N*_area_ than plants in low-irradiance climates. The third RDA axis explained little trait variation.

**Table 2:** Trait loadings, eigenvalues, and the percentage of trait variation explained by successive RDA axes (constrained by climate) and residual principal components.

	RDA1	RDA2	RDA3	PC1	PC2	PC3
ln SLA	-0.118	-**0**.**699**	**0**.**706**	-0.600	0.150	0.786
ln *N*_area_	0.282	**0**.**658**	**0**.**699**	0.733	-0.290	0.615
ln LAI	-**0**.**952**	0.281	0.120	-0.320	-0.945	-0.064
**Eigenvalue**	1.393	0.047	0.000	0.437	0.211	0.097
**Explained (%)**	62.640	2.217	0.000	20.090	9.937	4.594
**Cumulative (%)**	62.640	64.860	64.860	85.470	95.406	100


*Loadings >0.5 in magnitude are shown in bold.*

**FIGURE 4:**
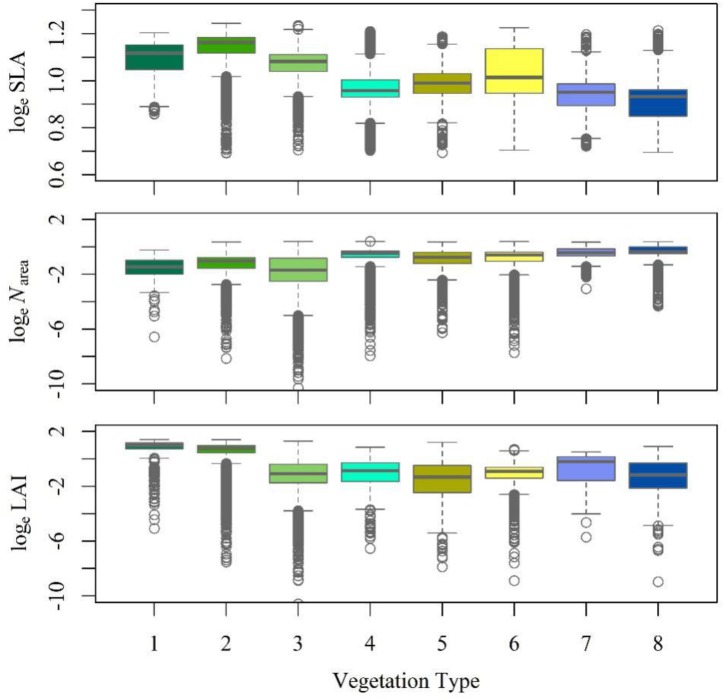
Trait statistics for each vegetation type. The box depicts the 25th, 50th, and 75th percentiles, and the top and bottom lines stand for the range (the whiskers). The gray circles stand for the outliers. (1) Tropical forest complex; (2) Subtropical forest complex; (3) Temperate forest complex; (4) Boreal and alpine forests; (5) Temperate scrub; (6) Temperate steppe; (7) Alpine steppe; and (8) Tundra.

The RDA showed that climate was the major determinant of trait variation for most of the traits examined. At the species level, the calibrated accuracy (adjusted R^2^ between observed and predicted values) was satisfactory at 0.16 for SLA, 0.3 for *N*_area_, and 0.84 for LAI (Supplementary Figure S2). The average adjusted R^2^ across traits was 0.43, which was higher than that obtained by Yang et al. (2019). Once the climatic variables were corrected for, we could predict the trait variation based on the trait–climate RDA relationships. We predicted the historical trait distributions with the climatic data from 2006 to 2014 (Supplementary Figure S3). Classified by vegetation types (Figure 4), tropical, subtropical and temperate forests had high SLA values, while tundra and alpine steppe usually had low SLA values. Alpine steppe, tundra and temperate steppe had higher *N*_area_ values, and tropical and subtropical forests had lower *N*_area_ values. Tropical and subtropical forests had a higher LAI, while temperate steppe and alpine steppe had a lower LAI. The vegetation types were characterized by different trait combinations, which make it possible to classify trait prediction values into different vegetation types with the help of GMM.

### Vegetation Distributions in the Three RCPs

For the GMM-based discriminant analysis, the distribution of vegetation derived from a natural vegetation map, together with the predicted historical trait patterns, were used to train the GMM classifier. Once the GMM density function was confirmed, the probability layers of each vegetation type were obtained according to the Gaussian density functions. The SLA-*N*_area_-LAI combination had a high accuracy in predicting vegetation distributions, and its overall accuracy was 75.14% and kappa coefficient was 69.27%. Desert and water (no vegetation region) were masked in this study. The optimal classification by the GMM and its comparison with a natural vegetation map are shown in Supplementary Figure S4. The good performance of the GMM made it possible to predict the vegetation distributions under different climate conditions.

**FIGURE 5:**
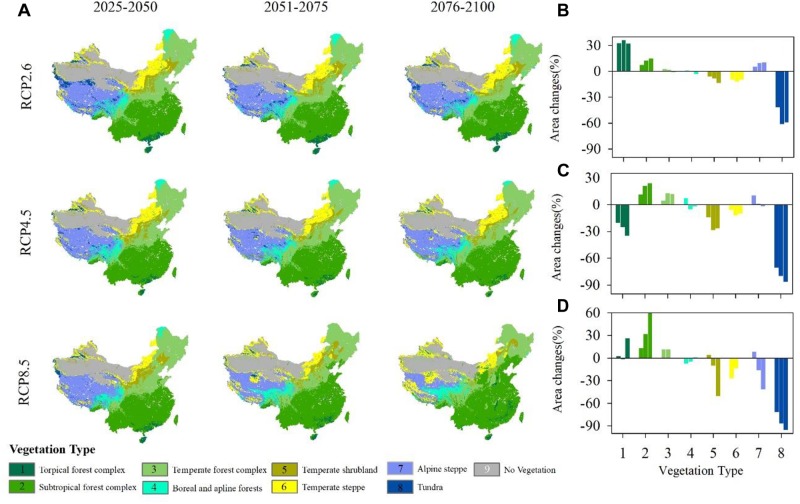
**(A)** Projected vegetation patterns under different representative concentration pathways (RCPs) during three different periods. **(B–D)** Proportion of vegetation area changes under RCP2.6, RCP4.5 and RCP8.5 compared with an historical vegetation map (Supplementary Figure S4A). (1) Tropical forest complex; (2) Subtropical forest complex; (3) Temperate forest complex; (4) Boreal and alpine forests; (5) Temperate scrub; (6) Temperate steppe; (7) Alpine steppe; (8) Tundra; and (9) No vegetation (masked).

Vegetation exhibits differently responses to climate change (Figure 5A). Under RCP2.6, the area of the tropical forest complex increases in the three periods (Figure 5B). Compared with the current vegetation distributions (Supplementary Figure S4A), the subtropical forest complex also shows an increasing trend during the three periods, although as time passes, the increase weakens. Similar to the temperate forest complex, the area of boreal and alpine forests changes little compared with the historical vegetation distributions. Temperate scrub and temperate steppe decrease slightly with time. Alpine steppe expands north, causing rapid tundra shrinkage.

Under RCP4.5, the tropical forest complex shows an obvious deceasing trend (Figure 5C) and is replaced by the subtropical forest complex. Both the subtropical forest complex and temperate forest shift north show an increasing trend in the area. Boreal forest shrinks as time passes and alpine forest first expands and then decreases in area from 2051 to 2075. Temperate scrub and temperate steppe shrink during all three periods. The area of temperate steppe changes little during the three periods. Similar to the patterns observed under RCP2.6, alpine steppe shifts north, causing tundra to shrink quickly and shift north.

Under RCP8.5, tropical forest shifts north, although its area changes little. From 2076 to 2100, the area of tropical forest increases by nearly 30% (Figure 5D). The subtropical forest complex shifts north and always expands rapidly. Temperate forest also expands, but its rate of increase is much lower than that of the tropical forest complex. Boreal forest, which is located in northeastern China, almost disappears in this scenario, although the alpine forest occupies a larger area on the Tibetan Plateau, causing the shrinkage of alpine steppe and tundra.

Among the three climatic scenarios, the subtropical forest complex, temperate forest and alpine steppe all show an increasing trend, while temperate scrub, temperate steppe and tundra show a decreasing trend. The most obvious increase occurred in the subtropical forest because it is broadly adapted to warm and humid climatic conditions, while tundra is the most sensitive vegetation type under such conditions. The most dramatic changes in vegetation distributions occur under the RCP8.5, for which the average rate of change reaches 25.20%, followed by RCP4.5 (22.10%) and RCP2.6 (16.50%).

## Discussion

### Linking the Trait Covariations Into Changes of Vegetation Distribution

Our simulations focused on trait variations (especially trait covariations) and vegetation distributions. We first predicted the trait covariations based on the trait–climate corresponding relationships and then classified the trait combinations into different vegetation types. This method had been validated by several studies (van Bodegom et al., 2014; Yang et al., 2016), and it has been improved in two aspects: one was that trait covariations were used in this study to provide more information about plant strategies, e.g., tundra vegetation simultaneously had the highest *N*_area_ and low SLA, indicating its high photosynthetic capacity and expensive investment in leaf construction; the other aspect was that the GMM could quantify the relationships between continuous trait changes and vegetation types and improve the classification accuracy for each vegetation type. Previous studies have focused on vegetation sensitivity under future climate scenarios and our results showed high consistency with these former studies (Wang et al., 2013; Yang et al., 2016); for example, both tropical forests and subtropical forests shifted to the north under the scenarios in which temperature increased, and the area of the boreal forest and tundra decreased. Although the vegetation distributions and PFTs were not necessary in the process of ecological models, they were still important outputs of the vegetation dynamic model.

### Ecological and Modeling Significance of Leaf Traits

Our analyses of leaf traits, including the SLA, *N*_area_, and LAI, could be treated as two important dimensions of trait covariation (Yang et al., 2019). The three plant traits used in this study have important ecological and modeling significance. SLA and *N*_area_ are included in the universal leaf economic spectrum. SLA is an essential variable in vegetation models because it determines the relationship between the LA available for light interception and photosynthesis and the amount of photosynthate required for leaf construction (Niklas et al., 2007). Compared with the leaf N concentration per unit mass (*N*_mass_), *N_area_* can be expressed more accurately as the sum of a metabolic component proportional to photosynthetic capacity and a structural component proportional to the leaf mass per area (LMA, LMA = 1/SLA) (Dong et al., 2017). *N_area_* is a key variable for modeling because it determines the N demand of leaf construction. This variable is represented in many recent vegetation models that include an interactive N cycle (Zaehle et al., 2014; Stocker et al., 2016). The LES reflects the linkage between high construction costs and long payback times of leaves with low SLA (McMurtrie and Dewar, 2011; Funk and Cornwell, 2013). More broadly, the LES is a universal feature of functional diversity within communities (see e.g., Hallik et al., 2009; Pierce et al., 2013) and as such, should be represented in models.

The LAI, which is equally significant to the LA, is expected to increase with precipitation and temperature due to energy-balance constraints (Wright et al., 2017). According to these constraints, leaf temperature is usually lower than air temperature under warm, well-watered, mid-day conditions (*T*_air_ between 25 and 30°C); otherwise, leaf temperature is higher than air temperature. At night, leaf temperature is usually lower than air temperature. By specifying the lower and upper thermal limits for leaf damage, we can predict the maximum LA in any climate. LA is an important target for modeling because (a) it contributes to the determination of the temperature at which photosynthesis, respiration and evaporation from the leaf surface take place, and (b) it climatically determines shifts in leaf size that should be linked to major changes in community composition. Three traits are co-located and present trait covariations effectively, which is important information for balancing traits in vegetation modeling.

### Challenges and Future Directions

This work highlights two challenges for modeling. The first is the “challenge of predictability” – the extent to which trait values can be predicted from independent information, including environmental factors and/or others. Recent research has mainly focused on the prediction of community-mean trait values. In fact, some trait variation, even in such a climatically wide-ranging data set, is not predictable by climate alone, and some other factors, such as site microclimate, life form, and family (phylogeny), are also important contributors to the trait variations (Yang et al., 2019). Nonetheless, climate emerges as a powerful control that allows empirical trait–climate relationships derived from data in China to be applied globally (Yang et al., 2019).

The second modeling challenge is the “challenge of functional diversity.” Trait-based models can represent the co-existence of multiple trait combinations. Moreover, this diversity confers increased resilience in model communities in the face of environmental change (Sakschewski et al., 2016). The challenge is to find a generally applicable method to specify the range of allowable trait combinations that is consistent with observed patterns of trait variation within sites. Ultimately, vegetation models should be able to predict, for example, experimentally determined relationships between species diversity and ecosystem function (e.g., Isbell et al., 2015), although this potential has yet to be realized.

## Conclusion

In conclusion, trait-based vegetation modeling provides a promising route toward ecosystem and land-surface models that are “reliable, robust, and realistic” (Prentice et al., 2015), and it can tackle a wider range of scientific questions than current models. Field measurements of key traits are valuable in providing information for trait-based model development, although despite the availability of large plant-trait data compilations (e.g., Kattge et al., 2011), the number of sites that include all of any specified set of plant traits is often disappointingly small because different groups typically collect data on different sets of traits. A limited amount of comparative work has been performed, such as on photosynthetic traits that are particularly important for vegetation modeling. Moreover, there remains a need for more extensive and co-located collections and analyses of plant functional traits (notably, stem hydraulic properties), which may be equally important for functional ecology and vegetation modeling.

## Data Availability

All datasets for this study are included in the manuscript and the Supplementary Files.

## Author Contributions

YY and PZ designed the modeling methods. JZ, HW, BW, SS, ML, LW, and ZP carried out the data analyses. YY wrote the first draft of the manuscript. QZ and CP provided additional advice on the analysis. All authors provided inputs on the final draft of the manuscript.

## Conflict of Interest Statement

The authors declare that the research was conducted in the absence of any commercial or financial relationships that could be construed as a potential conflict of interest.

## Footnotes

1 https://esgf-node.llnl.gov/projects/cmip5/
