# Supplementary material for: Trait-Based Climate Change Predictions of Vegetation Sensitivity and Distribution in China
Source: Front Plant Sci. 2019 Jul 12;10:908. doi: 10.3389/fpls.2019.00908 (PMC6640191; doi:10.3389/fpls.2019.00908)
Supplement: Supplementary file 1 [file Data_Sheet_1.docx]

**SUPPORTING INFORMATION**

**Trait‐based climate change predictions of vegetation sensitivity and distribution in China**

Yanzheng Yang^1,2^, Jun Zhao^1,2^, Pengxiang Zhao^1, *^, Hui Wang^1^, Boheng Wang^1^, Shaofeng Su^1^, Mingxu Li^1^, Liming Wang^3^, Qiuan Zhu^1^, Zhiyong Pang^4^, Changhui Peng^1,5^

^1^ College of Forestry, Northwest A&F University, Yangling, Shaanxi 712100, China

^2^ State Key Laboratory of Urban and Regional Ecology, Research Center for Eco-environmental Sciences, Chinese Academy of Sciences, Beijing 100085, China

^3^ State Key Laboratory of Hydroscience and Engineering, Department of Hydraulic Engineering, Tsinghua University, Beijing 100084, China

^4^ Institute of Surface-Earth System Science, Tianjin University, Tianjin, 300072, China

^5^ Department of Biology Sciences, Institute of Environment Sciences, University of Quebec at Montreal, Montreal, Canada

***Correspondence: Pengxiang Zhao** [zhaopengxiang@nwsuaf.edu.cn](mailto:zhaopengxiang@nwsuaf.edu.cn)

To be submitted to “***Frontiers in Plant Science***”

**Figure S1** Simulation steps


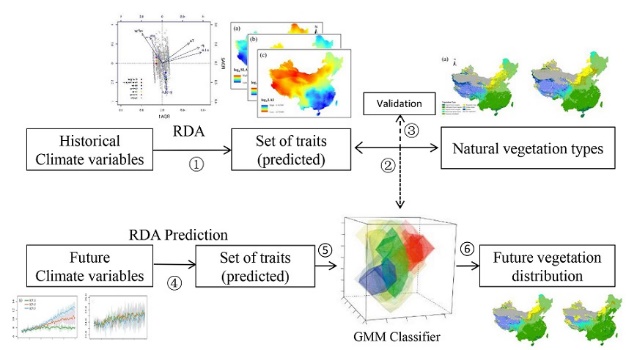


**Figure S2** Calibration of the predictive model of trait-climate RDA relationships in China. The traits are SLA: specific leaf area; *N*_area_: leaf nitrogen per unit area; and LAI: leaf area index.


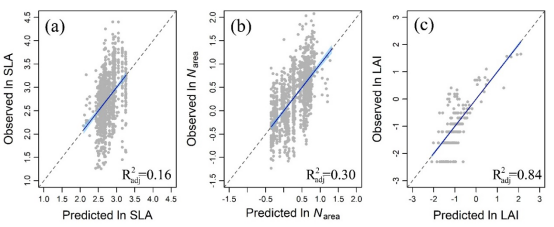


**Figure S3** Trait patterns predicted by the RDA trait-climate relationships. The traits are SLA: specific leaf area; *N*_area_: leaf nitrogen per unit area; and LAI: leaf area index.


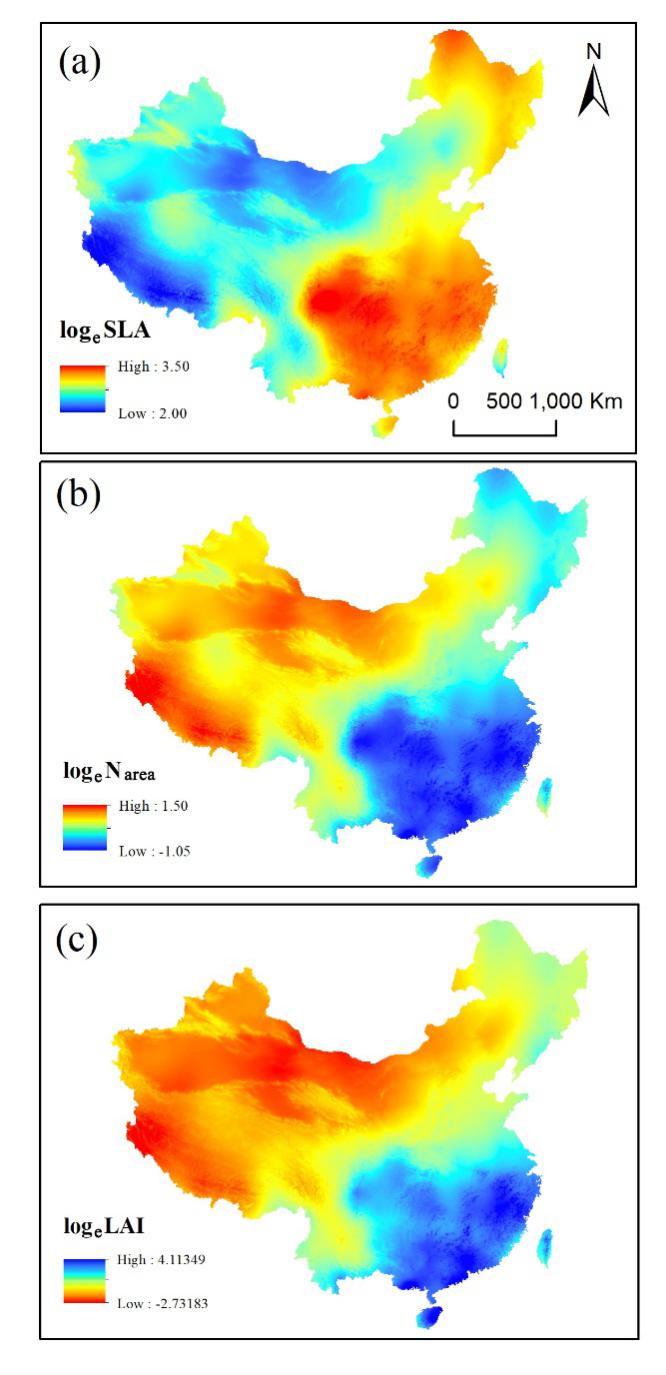


**Figure S4** Comparisons of the natural vegetation map (a) with classification results from the GMM classifier (b).1: Tropical forest complex; 2: Subtropical forest complex; 3: Temperate forest complex; 4: Boreal and alpine forests; 5: Temperate scrub; 6: Temperate steppe; 7: Alpine steppe; 8: Tundra; and 9: No vegetation (masked).


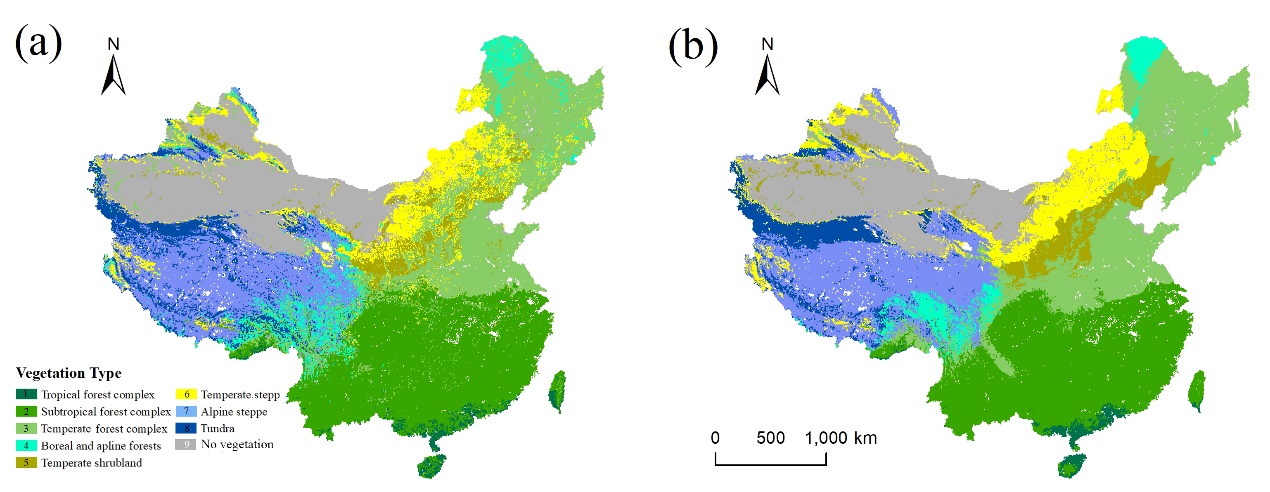


**Table S1** Basic information on the CMIP5 climate models used in this study

| Models | Institutions and countries | Resolution |
| --- | --- | --- |
| IPSL-CM5A-MR | Institute Pierre Simon Laplace, France | 1.27°×2.5° |
| MPI-ESM-MR | Max Planck Institute for Meteorology, Germany | 1.875°×1.85° |
| NorESM1-M | Norwegian Climate Centre, Norway | 2.5 °×1.9° |

**Table S2** Description of the sampling sites

| **Site name** | **Longitude** | **Latitude** | **Elevation** | **No. of species** | **Vegetation type** | **region cate** | **Source** |
| --- | --- | --- | --- | --- | --- | --- | --- |
| NECTS03 | 129.78 | 43.02 | 136 | 17 | temperate steppe | Changbai | Wang et al 2018 |
| NECTS04 | 130.08 | 42.98 | 114 | 15 | temperate steppe | Changbai | Wang et al 2018 |
| NECTS05 | 131.15 | 43.30 | 289 | 22 | temperate steppe | Changbai | Wang et al 2018 |
| NECTS06 | 131.00 | 43.12 | 244 | 17 | temperate steppe | Changbai | Wang et al 2018 |
| NECTS07 | 129.67 | 43.39 | 224 | 13 | temperate steppe | Changbai | Wang et al 2018 |
| NECTS08 | 128.64 | 43.25 | 601 | 10 | temperate steppe | Changbai | Wang et al 2018 |
| NECTS09 | 127.03 | 43.73 | 390 | 19 | temperate steppe | Changbai | Wang et al 2018 |
| NECTS10 | 125.68 | 43.81 | 252 | 11 | temperate steppe | Changbai | Wang et al 2018 |
| NECTS11 | 123.51 | 44.59 | 146 | 10 | temperate steppe | Changbai | Wang et al 2018 |
| NECTS12 | 123.27 | 44.43 | 150 | 6 | temperate steppe | Changbai | Wang et al 2018 |
| NECTS01 | 118.48 | 42.88 | 1024 | 7 | temperate steppe | Inner Mongolia | Wang et al 2018 |
| NECTS02 | 119.02 | 43.64 | 781 | 13 | temperate steppe | Inner Mongolia | Wang et al 2018 |
| NECTS13 | 121.84 | 43.60 | 203 | 10 | temperate steppe | Inner Mongolia | Wang et al 2018 |
| NECTS14 | 121.77 | 44.12 | 202 | 5 | temperate steppe | Inner Mongolia | Wang et al 2018 |
| NECTS15 | 120.55 | 44.39 | 448 | 10 | temperate steppe | Inner Mongolia | Wang et al 2018 |
| NECTS16 | 120.37 | 44.22 | 372 | 10 | temperate steppe | Inner Mongolia | Wang et al 2018 |
| NECTS17 | 119.38 | 43.88 | 601 | 10 | temperate steppe | Inner Mongolia | Wang et al 2018 |
| NECTS18 | 119.12 | 43.76 | 729 | 12 | temperate steppe | Inner Mongolia | Wang et al 2018 |
| NECTS19 | 118.49 | 43.34 | 707 | 6 | temperate steppe | Inner Mongolia | Wang et al 2018 |
| NECTS20 | 117.76 | 43.19 | 889 | 9 | temperate steppe | Inner Mongolia | Wang et al 2018 |
| NECTS21 | 117.24 | 43.22 | 1259 | 11 | temperate steppe | Inner Mongolia | Wang et al 2018 |
| NECTS22 | 116.89 | 43.39 | 1267 | 7 | temperate steppe | Inner Mongolia | Wang et al 2018 |
| NECTS23 | 116.68 | 43.55 | 1261 | 12 | temperate steppe | Inner Mongolia | Wang et al 2018 |
| NECTS24 | 116.64 | 43.69 | 1211 | 9 | temperate steppe | Inner Mongolia | Wang et al 2018 |
| NECTS25 | 116.31 | 43.91 | 1199 | 11 | temperate steppe | Inner Mongolia | Wang et al 2018 |
| NECTS26 | 115.32 | 43.90 | 1196 | 10 | temperate steppe | Inner Mongolia | Wang et al 2018 |
| NECTS27 | 114.61 | 43.94 | 1123 | 8 | temperate steppe | Inner Mongolia | Wang et al 2018 |
| NECTS28 | 113.83 | 43.83 | 1166 | 8 | temperate steppe | Inner Mongolia | Wang et al 2018 |
| NECTS29 | 113.36 | 43.80 | 1017 | 6 | temperate steppe | Inner Mongolia | Wang et al 2018 |
| NECTS30 | 112.59 | 43.72 | 974 | 9 | temperate steppe | Inner Mongolia | Wang et al 2018 |
| NECTS31 | 112.17 | 43.63 | 999 | 8 | temperate steppe | Inner Mongolia | Wang et al 2018 |
| NECTS32 | 111.92 | 43.66 | 1005 | 8 | temperate steppe | Inner Mongolia | Wang et al 2018 |
| NECTS33 | 111.89 | 43.65 | 1017 | 7 | temperate steppe | Inner Mongolia | Wang et al 2018 |
| Mohe Flux | 122.34 | 53.47 | 290 | 20 | boreal forest | Mohe | Wang et al 2018 |
| Mohe Ghost-train | 122.34 | 53.46 | 325 | 20 | boreal forest | Mohe | Wang et al 2018 |
| Mohe Hilltop | 122.25 | 53.39 | 638 | 20 | boreal forest | Mohe | Wang et al 2018 |
| Qinling Mixed Forest | 108.44 | 33.44 | 1514 | 33 | tempeature forest | Qinling | Wang et al 2018 |
| Ailaoshan Dwarf | 101.03 | 24.54 | 2637 | 20 | tropical and subtropical forest | XSBN | Wang et al 2018 |
| Ailaoshan Flux | 101.03 | 24.54 | 2394 | 36 | tropical and subtropical forest | XSBN | Wang et al 2018 |
| Ailaoshan Mid | 100.99 | 24.50 | 2056 | 34 | tropical and subtropical forest | XSBN | Wang et al 2018 |
| Long Ling 1 | 101.58 | 21.62 | 1034 | 36 | tropical and subtropical forest | XSBN | Wang et al 2018 |
| Mandan Shrub | 101.85 | 23.69 | 758 | 32 | tropical and subtropical forest | XSBN | Wang et al 2018 |
| Mandan Wood | 101.86 | 23.69 | 772 | 39 | tropical and subtropical forest | XSBN | Wang et al 2018 |
| Mengla 1 Rainforest | 101.58 | 21.61 | 668 | 42 | tropical and subtropical forest | XSBN | Wang et al 2018 |
| Mengla 2 Midslope | 101.58 | 21.62 | 828 | 19 | tropical and subtropical forest | XSBN | Wang et al 2018 |
| Unholy Mt | 101.24 | 21.98 | 1075 | 40 | tropical and subtropical forest | XSBN | Wang et al 2018 |
| XBTG Rainforest | 101.27 | 21.92 | 502 | 44 | tropical and subtropical forest | XSBN | Wang et al 2018 |
| NM-02 | 116.36 | 43.89 | 1206 | 2 | temperate steppe | Inner Mongolia | Geng et al 2017 |
| NM-03 | 116.49 | 43.83 | 1101 | 1 | temperate steppe | Inner Mongolia | Geng et al 2017 |
| NM-04 | 116.11 | 43.74 | 1016 | 3 | temperate steppe | Inner Mongolia | Geng et al 2017 |
| NM-10 | 117.04 | 45.59 | 907 | 3 | temperate steppe | Inner Mongolia | Geng et al 2017 |
| NM-11 | 117.92 | 45.70 | 0 | 3 | temperate steppe | Inner Mongolia | Geng et al 2017 |
| NM-12 | 119.30 | 47.65 | 0 | 5 | temperate steppe | Inner Mongolia | Geng et al 2017 |
| NM-13 | 118.60 | 48.41 | 0 | 3 | temperate steppe | Inner Mongolia | Geng et al 2017 |
| NM-14 | 120.06 | 49.40 | 890 | 3 | temperate steppe | Inner Mongolia | Geng et al 2017 |
| NM-16 | 119.34 | 49.30 | 844 | 4 | temperate steppe | Inner Mongolia | Geng et al 2017 |
| NM-17 | 118.46 | 49.49 | 776 | 4 | temperate steppe | Inner Mongolia | Geng et al 2017 |
| NM-18 | 117.30 | 49.51 | 703 | 1 | temperate steppe | Inner Mongolia | Geng et al 2017 |
| NM-20 | 116.63 | 48.60 | 582 | 2 | temperate steppe | Inner Mongolia | Geng et al 2017 |
| NM-21 | 117.14 | 48.49 | 588 | 4 | temperate steppe | Inner Mongolia | Geng et al 2017 |
| NM-22 | 118.10 | 48.20 | 575 | 2 | temperate steppe | Inner Mongolia | Geng et al 2017 |
| NM-23 | 118.75 | 47.89 | 733 | 2 | temperate steppe | Inner Mongolia | Geng et al 2017 |
| NM-24 | 119.50 | 46.60 | 1060 | 3 | temperate steppe | Inner Mongolia | Geng et al 2017 |
| NM-26 | 118.54 | 46.27 | 857 | 5 | temperate steppe | Inner Mongolia | Geng et al 2017 |
| NM-27 | 118.26 | 46.16 | 919 | 2 | temperate steppe | Inner Mongolia | Geng et al 2017 |
| NM-28 | 116.91 | 45.41 | 877 | 3 | temperate steppe | Inner Mongolia | Geng et al 2017 |
| NM-29 | 116.13 | 44.93 | 873 | 3 | temperate steppe | Inner Mongolia | Geng et al 2017 |
| NM-33 | 115.31 | 43.90 | 1187 | 5 | temperate steppe | Inner Mongolia | Geng et al 2017 |
| NM-34 | 115.31 | 43.90 | 1200 | 3 | temperate steppe | Inner Mongolia | Geng et al 2017 |
| NM-35 | 114.22 | 43.88 | 1021 | 3 | temperate steppe | Inner Mongolia | Geng et al 2017 |
| NM-38 | 112.07 | 43.63 | 952 | 2 | temperate steppe | Inner Mongolia | Geng et al 2017 |
| NM-39 | 111.91 | 43.36 | 1037 | 1 | temperate steppe | Inner Mongolia | Geng et al 2017 |
| NM-42 | 108.76 | 41.51 | 1299 | 2 | temperate steppe | Inner Mongolia | Geng et al 2017 |
| NM-43 | 108.83 | 41.50 | 1286 | 3 | temperate steppe | Inner Mongolia | Geng et al 2017 |
| NM-44 | 108.25 | 41.87 | 1346 | 3 | temperate steppe | Inner Mongolia | Geng et al 2017 |
| NM-45 | 108.55 | 41.82 | 1527 | 3 | temperate steppe | Inner Mongolia | Geng et al 2017 |
| NM-46 | 108.64 | 39.76 | 1425 | 4 | temperate steppe | Inner Mongolia | Geng et al 2017 |
| NM-47 | 108.20 | 39.32 | 1437 | 3 | temperate steppe | Inner Mongolia | Geng et al 2017 |
| NM-48 | 107.62 | 38.91 | 1217 | 3 | temperate steppe | Inner Mongolia | Geng et al 2017 |
| NM-49 | 107.26 | 38.41 | 1458 | 4 | temperate steppe | Inner Mongolia | Geng et al 2017 |
| NM-JinDongwu | 116.44 | 45.07 | 858 | 2 | temperate steppe | Inner Mongolia | Geng et al 2017 |
| NM-Lanqi-1 | 116.13 | 43.26 | 1297 | 4 | temperate steppe | Inner Mongolia | Geng et al 2017 |
| NM-Lanqi-2 | 115.96 | 42.97 | 1231 | 5 | temperate steppe | Inner Mongolia | Geng et al 2017 |
| NM-Lanqi-3 | 115.92 | 42.16 | 1299 | 4 | temperate steppe | Inner Mongolia | Geng et al 2017 |
| NM-Lanqi-4 | 115.88 | 42.83 | 1305 | 3 | temperate steppe | Inner Mongolia | Geng et al 2017 |
| NM-Lymus | 116.35 | 43.55 | 1259 | 3 | temperate steppe | Inner Mongolia | Geng et al 2017 |
| NM-Stipa | 116.55 | 43.54 | 1178 | 2 | temperate steppe | Inner Mongolia | Geng et al 2017 |
| NM-Xilinhe | 116.66 | 43.62 | 1168 | 2 | temperate steppe | Inner Mongolia | Geng et al 2017 |
| GS-50 | 102.44 | 35.10 | 3107 | 2 | Alpine steppe | Tibet | Geng et al 2017 |
| GS-51 | 102.89 | 34.97 | 2996 | 4 | Alpine steppe | Tibet | Geng et al 2017 |
| GS-52 | 102.83 | 34.90 | 3241 | 3 | Alpine steppe | Tibet | Geng et al 2017 |
| GS-53 | 102.34 | 34.49 | 3542 | 3 | Alpine steppe | Tibet | Geng et al 2017 |
| GS-54 | 102.34 | 34.28 | 3491 | 2 | Alpine steppe | Tibet | Geng et al 2017 |
| GS-55 | 102.49 | 34.70 | 3273 | 6 | Alpine steppe | Tibet | Geng et al 2017 |
| GS-56 | 102.51 | 34.71 | 3311 | 3 | Alpine steppe | Tibet | Geng et al 2017 |
| GS-57 | 102.09 | 34.05 | 3567 | 2 | Alpine steppe | Tibet | Geng et al 2017 |
| Q01 | 100.89 | 36.32 | 3277 | 1 | Alpine steppe | Tibet | Geng et al 2017 |
| Q02 | 100.46 | 36.12 | 2934 | 3 | Alpine steppe | Tibet | Geng et al 2017 |
| Q04 | 100.22 | 36.00 | 3078 | 2 | Alpine steppe | Tibet | Geng et al 2017 |
| Q06 | 100.23 | 35.76 | 3184 | 6 | Alpine steppe | Tibet | Geng et al 2017 |
| Q07 | 100.49 | 35.57 | 3304 | 3 | Alpine steppe | Tibet | Geng et al 2017 |
| Q09 | 100.93 | 35.35 | 3253 | 2 | Alpine steppe | Tibet | Geng et al 2017 |
| Q10 | 100.77 | 35.08 | 3565 | 4 | Alpine steppe | Tibet | Geng et al 2017 |
| Q11 | 100.82 | 34.86 | 3650 | 3 | Alpine steppe | Tibet | Geng et al 2017 |
| Q12 | 100.40 | 34.45 | 3938 | 4 | Alpine steppe | Tibet | Geng et al 2017 |
| Q13 | 100.22 | 34.53 | 3727 | 12 | Alpine steppe | Tibet | Geng et al 2017 |
| Q16 | 99.93 | 34.47 | 3930 | 7 | Alpine steppe | Tibet | Geng et al 2017 |
| Q18 | 98.97 | 34.84 | 4518 | 4 | Alpine steppe | Tibet | Geng et al 2017 |
| Q21 | 99.18 | 35.36 | 4158 | 2 | Alpine steppe | Tibet | Geng et al 2017 |
| Q22 | 99.39 | 35.43 | 4002 | 1 | Alpine steppe | Tibet | Geng et al 2017 |
| Q23 | 99.48 | 35.44 | 4089 | 2 | Alpine steppe | Tibet | Geng et al 2017 |
| Q24 | 98.58 | 34.99 | 4297 | 2 | Alpine steppe | Tibet | Geng et al 2017 |
| Q25 | 98.45 | 34.85 | 4219 | 3 | Alpine steppe | Tibet | Geng et al 2017 |
| Q26 | 98.25 | 34.88 | 4229 | 2 | Alpine steppe | Tibet | Geng et al 2017 |
| Q30 | 97.99 | 34.58 | 4278 | 2 | Alpine steppe | Tibet | Geng et al 2017 |
| Q31 | 97.66 | 34.20 | 5249 | 4 | Alpine steppe | Tibet | Geng et al 2017 |
| Q32 | 97.02 | 33.76 | 4589 | 3 | Alpine steppe | Tibet | Geng et al 2017 |
| Q34 | 96.37 | 33.97 | 4229 | 3 | Alpine steppe | Tibet | Geng et al 2017 |
| Q35 | 96.20 | 34.10 | 4363 | 6 | Alpine steppe | Tibet | Geng et al 2017 |
| Q37 | 95.80 | 34.14 | 4226 | 2 | Alpine steppe | Tibet | Geng et al 2017 |
| Q38 | 95.70 | 33.95 | 4161 | 2 | Alpine steppe | Tibet | Geng et al 2017 |
| Q39 | 95.88 | 33.73 | 4264 | 2 | Alpine steppe | Tibet | Geng et al 2017 |
| Q40 | 96.01 | 33.60 | 4330 | 3 | Alpine steppe | Tibet | Geng et al 2017 |
| Q41 | 96.36 | 33.28 | 4292 | 1 | Alpine steppe | Tibet | Geng et al 2017 |
| Q43 | 96.74 | 33.11 | 4238 | 2 | Alpine steppe | Tibet | Geng et al 2017 |
| Q44 | 96.91 | 33.02 | 3901 | 3 | Alpine steppe | Tibet | Geng et al 2017 |
| Q47 | 96.74 | 32.90 | 4286 | 4 | Alpine steppe | Tibet | Geng et al 2017 |
| Q48 | 96.56 | 32.59 | 3958 | 5 | Alpine steppe | Tibet | Geng et al 2017 |
| X01 | 96.53 | 31.97 | 4167 | 4 | Alpine steppe | Tibet | Geng et al 2017 |
| X02 | 96.39 | 32.00 | 4191 | 5 | Alpine steppe | Tibet | Geng et al 2017 |
| X03 | 96.51 | 31.10 | 4631 | 10 | Alpine steppe | Tibet | Geng et al 2017 |
| X04 | 94.96 | 31.70 | 4336 | 8 | Alpine steppe | Tibet | Geng et al 2017 |
| X06 | 93.79 | 31.84 | 4014 | 3 | Alpine steppe | Tibet | Geng et al 2017 |
| X08 | 93.54 | 31.85 | 4475 | 2 | Alpine steppe | Tibet | Geng et al 2017 |
| X09 | 93.14 | 31.93 | 4478 | 1 | Alpine steppe | Tibet | Geng et al 2017 |
| X10 | 92.90 | 31.84 | 4307 | 2 | Alpine steppe | Tibet | Geng et al 2017 |
| X12 | 92.87 | 31.83 | 4287 | 3 | Alpine steppe | Tibet | Geng et al 2017 |
| X17 | 91.69 | 31.10 | 4758 | 1 | Alpine steppe | Tibet | Geng et al 2017 |
| X19 | 90.81 | 30.31 | 4328 | 1 | Alpine steppe | Tibet | Geng et al 2017 |
| XX01 | 90.42 | 29.26 | 3667 | 1 | Alpine steppe | Tibet | Geng et al 2017 |
| XX02 | 89.95 | 29.33 | 3706 | 1 | Alpine steppe | Tibet | Geng et al 2017 |
| XX3 | 86.83 | 28.19 | 5100 | 2 | Alpine steppe | Tibet | Geng et al 2017 |
| XX4 | 86.84 | 28.30 | 4622 | 2 | Alpine steppe | Tibet | Geng et al 2017 |
| XX5 | 87.07 | 28.51 | 5242 | 4 | Alpine steppe | Tibet | Geng et al 2017 |
| XX6 | 88.15 | 29.15 | 4080 | 1 | Alpine steppe | Tibet | Geng et al 2017 |
| XJ01 | 86.49 | 43.81 | 1418 | 2 | temperate steppe | Xinjiang | Geng et al 2017 |
| XJ02 | 86.77 | 43.76 | 1105 | 1 | temperate steppe | Xinjiang | Geng et al 2017 |
| XJ03 | 85.78 | 45.94 | 1259 | 2 | temperate steppe | Xinjiang | Geng et al 2017 |
| XJ04 | 85.44 | 43.95 | 1356 | 1 | temperate steppe | Xinjiang | Geng et al 2017 |
| XJ05 | 85.32 | 43.96 | 1536 | 2 | temperate steppe | Xinjiang | Geng et al 2017 |
| XJ06 | 84.83 | 44.14 | 1424 | 2 | temperate steppe | Xinjiang | Geng et al 2017 |
| XJ07 | 84.81 | 44.09 | 1887 | 3 | temperate steppe | Xinjiang | Geng et al 2017 |
| XJ08 | 84.40 | 43.66 | 2505 | 3 | temperate steppe | Xinjiang | Geng et al 2017 |
| XJ09 | 84.40 | 43.53 | 2627 | 2 | temperate steppe | Xinjiang | Geng et al 2017 |
| XJ10 | 84.42 | 43.45 | 3267 | 1 | temperate steppe | Xinjiang | Geng et al 2017 |
| XJ11 | 84.37 | 43.16 | 2717 | 2 | temperate steppe | Xinjiang | Geng et al 2017 |
| XJ13 | 85.47 | 43.11 | 2885 | 2 | temperate steppe | Xinjiang | Geng et al 2017 |
| XJ16 | 83.28 | 42.45 | 2474 | 1 | temperate steppe | Xinjiang | Geng et al 2017 |
| XJ17 | 81.31 | 41.94 | 2471 | 1 | temperate steppe | Xinjiang | Geng et al 2017 |
| XJ18 | 81.36 | 41.92 | 1996 | 1 | temperate steppe | Xinjiang | Geng et al 2017 |
| XJ19 | 80.65 | 41.81 | 2358 | 1 | temperate steppe | Xinjiang | Geng et al 2017 |
| XJ20 | 77.91 | 39.92 | 1205 | 2 | temperate steppe | Xinjiang | Geng et al 2017 |
| XJ21 | 75.32 | 36.77 | 2535 | 2 | temperate steppe | Xinjiang | Geng et al 2017 |
| XJ22 | 75.05 | 38.42 | 3664 | 2 | temperate steppe | Xinjiang | Geng et al 2017 |
| XJ23 | 75.18 | 37.92 | 3101 | 2 | temperate steppe | Xinjiang | Geng et al 2017 |
| XJ24 | 74.93 | 38.30 | 3812 | 1 | temperate steppe | Xinjiang | Geng et al 2017 |
| XJ25 | 75.24 | 38.91 | 2591 | 2 | temperate steppe | Xinjiang | Geng et al 2017 |
| XJ26 | 75.57 | 39.20 | 1460 | 1 | temperate steppe | Xinjiang | Geng et al 2017 |
| XJ27 | 86.11 | 43.02 | 2992 | 2 | temperate steppe | Xinjiang | Geng et al 2017 |
| XJ28 | 84.90 | 43.15 | 2662 | 3 | temperate steppe | Xinjiang | Geng et al 2017 |
| XJ29 | 84.03 | 43.30 | 1523 | 1 | temperate steppe | Xinjiang | Geng et al 2017 |
| XJ30 | 83.72 | 43.40 | 1266 | 2 | temperate steppe | Xinjiang | Geng et al 2017 |
| XJ31 | 83.50 | 43.40 | 1100 | 2 | temperate steppe | Xinjiang | Geng et al 2017 |
| XJ32 | 83.17 | 43.38 | 1101 | 1 | temperate steppe | Xinjiang | Geng et al 2017 |
| XJ33 | 82.23 | 43.38 | 890 | 1 | temperate steppe | Xinjiang | Geng et al 2017 |
| XJ34 | 81.87 | 43.38 | 1466 | 2 | temperate steppe | Xinjiang | Geng et al 2017 |
| XJ35 | 81.68 | 43.50 | 1344 | 1 | temperate steppe | Xinjiang | Geng et al 2017 |
| XJ36 | 81.13 | 43.20 | 2005 | 2 | temperate steppe | Xinjiang | Geng et al 2017 |
| XJ37 | 81.37 | 43.15 | 1729 | 1 | temperate steppe | Xinjiang | Geng et al 2017 |
| XJ38 | 82.15 | 43.90 | 1064 | 2 | temperate steppe | Xinjiang | Geng et al 2017 |
| XJ39 | 80.97 | 44.35 | 1181 | 2 | temperate steppe | Xinjiang | Geng et al 2017 |
| XJ40 | 81.15 | 44.49 | 2126 | 1 | temperate steppe | Xinjiang | Geng et al 2017 |
| XJ41 | 81.22 | 44.50 | 2104 | 2 | temperate steppe | Xinjiang | Geng et al 2017 |
| XJ42 | 81.30 | 44.62 | 2101 | 1 | temperate steppe | Xinjiang | Geng et al 2017 |
| XJ43 | 81.15 | 45.02 | 1247 | 2 | temperate steppe | Xinjiang | Geng et al 2017 |
| XJ44 | 80.98 | 45.00 | 1428 | 2 | temperate steppe | Xinjiang | Geng et al 2017 |
| XJ45 | 81.25 | 44.87 | 1416 | 1 | temperate steppe | Xinjiang | Geng et al 2017 |
| XJ46 | 81.28 | 44.65 | 2106 | 1 | temperate steppe | Xinjiang | Geng et al 2017 |
| XJ47 | 81.47 | 44.62 | 2105 | 1 | temperate steppe | Xinjiang | Geng et al 2017 |
| XJ50 | 84.42 | 45.45 | 852 | 2 | temperate steppe | Xinjiang | Geng et al 2017 |
| XJ51 | 84.22 | 45.58 | 1140 | 1 | temperate steppe | Xinjiang | Geng et al 2017 |
| XJ52 | 83.90 | 45.50 | 1440 | 2 | temperate steppe | Xinjiang | Geng et al 2017 |
| XJ53 | 83.65 | 45.58 | 1820 | 1 | temperate steppe | Xinjiang | Geng et al 2017 |
| XJ54 | 83.57 | 45.80 | 1506 | 1 | temperate steppe | Xinjiang | Geng et al 2017 |
| XJ56 | 84.12 | 46.23 | 967 | 1 | temperate steppe | Xinjiang | Geng et al 2017 |
| XJ57 | 84.82 | 46.43 | 1113 | 1 | temperate steppe | Xinjiang | Geng et al 2017 |
| XJ58 | 84.63 | 45.92 | 1000 | 1 | temperate steppe | Xinjiang | Geng et al 2017 |
| XJ59 | 86.02 | 46.60 | 1046 | 2 | temperate steppe | Xinjiang | Geng et al 2017 |
| XJ60 | 86.10 | 46.70 | 1032 | 1 | temperate steppe | Xinjiang | Geng et al 2017 |
| XJ61 | 86.28 | 46.82 | 1142 | 1 | temperate steppe | Xinjiang | Geng et al 2017 |
| XJ62 | 86.52 | 46.97 | 1272 | 1 | temperate steppe | Xinjiang | Geng et al 2017 |
| XJ63 | 85.70 | 47.22 | 2136 | 2 | temperate steppe | Xinjiang | Geng et al 2017 |
| XJ64 | 86.17 | 47.28 | 1180 | 2 | temperate steppe | Xinjiang | Geng et al 2017 |
| XJ65 | 86.65 | 47.13 | 1125 | 1 | temperate steppe | Xinjiang | Geng et al 2017 |
| XJ67 | 86.92 | 48.80 | 1794 | 1 | temperate steppe | Xinjiang | Geng et al 2017 |
| XJ71 | 89.48 | 47.05 | 1085 | 1 | temperate steppe | Xinjiang | Geng et al 2017 |
| XJ72 | 89.37 | 46.87 | 1008 | 1 | temperate steppe | Xinjiang | Geng et al 2017 |
| XJ73 | 89.83 | 46.67 | 1103 | 1 | temperate steppe | Xinjiang | Geng et al 2017 |
| XJ77 | 90.07 | 44.25 | 733 | 2 | temperate steppe | Xinjiang | Geng et al 2017 |
| XJ78 | 90.62 | 43.65 | 1778 | 1 | temperate steppe | Xinjiang | Geng et al 2017 |
| XJ79 | 90.30 | 43.76 | 1422 | 1 | temperate steppe | Xinjiang | Geng et al 2017 |
| XJ80 | 93.67 | 43.35 | 2138 | 2 | temperate steppe | Xinjiang | Geng et al 2017 |
| XJ81 | 93.72 | 43.38 | 2120 | 1 | temperate steppe | Xinjiang | Geng et al 2017 |
| XJ84 | 93.70 | 43.28 | 2651 | 1 | temperate steppe | Xinjiang | Geng et al 2017 |
| XJ87 | 92.53 | 43.63 | 1737 | 1 | temperate steppe | Xinjiang | Geng et al 2017 |
| XJ88 | 92.30 | 43.70 | 2018 | 1 | temperate steppe | Xinjiang | Geng et al 2017 |
| XJ89 | 91.42 | 43.70 | 1670 | 1 | temperate steppe | Xinjiang | Geng et al 2017 |
| XJ92 | 87.18 | 43.53 | 1690 | 1 | temperate steppe | Xinjiang | Geng et al 2017 |
